# Integrated Multi-Omics Analysis Reveals the Involvement of Alanine, Aspartate and Glutamate Metabolism in Wheat Responses to Salt Stress

**DOI:** 10.3390/plants15182783

**Published:** 2026-09-11

**Authors:** Jixing Shang, Juncheng Wang, Xiaole Ma, Xingmao Li, Hong Zhang, Huajun Wang, Lirong Yao, Baochun Li

**Affiliations:** 1 College of Life Sciences and Technology, Gansu Agricultural University, Lanzhou 730070, China; sjxgslz@163.com; 2 State Key Lab of Aridland Crop Science, Gansu Key Lab of Crop Improvement and Germplasm Enhancement, Lanzhou 730070, China; wangjc@gsau.edu.cn (J.W.); maxl@gsau.edu.cn (X.M.); zhanghong@gsau.edu.cn (H.Z.); wanghj@gsau.edu.cn (H.W.); 3 College of Agronomy, Gansu Agricultural University, Lanzhou 730070, China; 4 Institute of Dryland Agriculture, Gansu Academy of Agricultural Sciences, Lanzhou 730070, China; lxm759@163.com

**Keywords:** wheat, salt stress, transcriptome, metabolome, integrative analysis

## Abstract

Salt stress is frequently encountered by plants and is a major constraint on crop production. Wheat (*Triticum aestivum* L.) is a staple cereal crop that is important for human health and food security. However, the response mechanisms of roots from different wheat varieties to salt stress remain unclear. In this study, we analyzed the complex response mechanisms of the salt-tolerant variety Longjian 114 (LJ114) and the salt-sensitive variety Chinese Spring (CS) to salt stress using integrated transcriptomic and metabolomic analyses. The results showed that LJ114 maintained better growth and exhibited higher antioxidant enzyme activities and greater osmotic adjustment capacity under salt stress. Differentially expressed genes in LJ114 were predominantly enriched in flavonoid biosynthesis, alpha-linolenic acid metabolism, and zeatin biosynthesis under salt stress. Metabolomic profiling identified 1366 differential metabolites, with LJ114 specifically enriched in tyrosine metabolism, tryptophan metabolism, glycerophospholipid metabolism, and fatty acid metabolism under salt stress. Integrated analyses further highlighted ABC transporters and alanine, aspartate, and glutamate metabolism as hub pathways, with notably higher *GAD* expression and GABA accumulation under salt stress. These findings suggest new insights into the molecular mechanisms underlying wheat salt tolerance and propose potential candidate targets for enhancing crop salinity tolerance.

## 1. Introduction

Soil salinization is a major abiotic stress that limits agricultural productivity and threatens food security [1]. Salt stress impairs plant growth and development through osmotic stress, ion toxicity, and oxidative damage, leading to reduced root vigor, impaired photosynthesis, decreased biomass, and ultimately yield loss [2,3,4]. Wheat (*Triticum aestivum* L.), a globally important staple crop, has relatively low salt tolerance and is particularly vulnerable during germination and the seedling stage [5,6]. Salt stress severely restricts root elongation, seedling establishment, and above-ground growth, negatively affecting subsequent yield formation [7,8]. Elucidating the molecular mechanisms that govern wheat responses to salt stress and identifying the associated genes, metabolites, and regulatory pathways is therefore essential for screening salt-tolerant germplasm and advancing molecular breeding.

Previous studies have shown that the adverse effects of salt stress on wheat primarily arise from osmotic imbalance, disruption of Na^+^/K^+^ homeostasis, excessive accumulation of reactive oxygen species, and membrane lipid peroxidation [9]. To adapt to saline conditions, plants restrict excessive Na^+^ uptake and its translocation to shoots, thereby maintaining ion homeostasis. They also accumulate osmolytes such as soluble sugars and proteins, enhance antioxidant defense systems, and activate signaling networks mediated by ABA, Ca^2+,^ and various transcription factor families [10,11,12]. Salt tolerance involves multiple biological processes, including antioxidant defense, ion homeostasis maintenance, hormone signal transduction, cell wall remodeling, carbon and nitrogen metabolism, and secondary metabolism, all strongly influenced by genetic background [7,13]. For instance, wheat reduces shoot Na^+^ accumulation through the *TaSPL6-D/TaHKT1;5-D* module [14]. *TaWRKY17* enhances antioxidant capacity and salt tolerance by modulating ABA/ROS-related pathways [15]. *TabZIP60* interacts with *TaCDPK30* in ABA biosynthesis-mediated salt stress responses, and certain *TaNAC* family members are associated with root salt tolerance regulation [12]. Transcription factor families such as WRKY, MYB, bHLH, ERF and NAC play important roles in salt stress responses by regulating ROS scavenging, osmotic adjustment, hormone signaling and secondary metabolism [16,17,18].

Salt tolerance in wheat is a complex quantitative trait governed by multiple genes, pathways and hierarchical networks. Phenotypic observations and physiological measurements alone cannot systematically elucidate its molecular basis [19,20]. Transcriptomics provides an effective approach for dissecting plant responses to salt stress. RNA-seq enables systematic identification of salt-induced differentially expressed genes, and weighted gene co-expression network analysis (WGCNA) can identify core gene modules associated with salt-tolerant phenotypes and physiological indicators. Chen et al. integrated RNA-seq, WGCNA, and association analysis to identify key modules and candidate genes in wheat roots responding to salt stress, demonstrating that root co-expression networks are crucial for regulating salt tolerance [20]. Wang et al. examined salt stress during germination and the seedling stage, revealing significant differences in gene expression patterns and co-expression modules between salt-tolerant and salt-sensitive cultivars, suggesting that different genotypes employ distinct strategies in response to salinity [7]. However, transcriptional changes alone cannot fully explain the development of salt-tolerant phenotypes. Ultimately, plant adaptation to salt stress depends on metabolite accumulation and metabolic pathway remodeling. Metabolomics provides a more direct reflection of phenotypic outcomes and can capture dynamic changes in osmotic adjustment, membrane lipid remodeling, and antioxidant metabolism [8]. It serves as a critical link between gene expression alterations and phenotypic responses [21,22]. In response to salt stress, plants typically accumulate amino acids, organic acids, soluble sugars, lipids, flavonoids, phenolic acids, and other secondary metabolites to maintain osmotic balance, scavenge reactive oxygen species, stabilize cellular membranes, and regulate energy supply [23]. Moreover, lipid and secondary metabolism are closely associated with salt tolerance. Salt stress alters membrane lipid composition and fluidity, while glycerophospholipid metabolism, fatty acid metabolism, α-linolenic acid metabolism, and the accumulation of flavonoids and phenylpropanoids enhance membrane stability and antioxidant capacity [8,24].

In recent years, integrative multi-omics analysis has become an important approach for elucidating the complex mechanisms governing plant responses to abiotic stress. Xu et al. combined transcriptomic and metabolomic analyses to identify significant changes in glycerolipid metabolism, phenylalanine metabolism, and glycerophospholipid metabolism in wheat under salt stress [25]. Yue et al. further demonstrated that both salt stress and combined stresses influence malondialdehyde accumulation, carbohydrate metabolism, amino acid metabolism and the tricarboxylic acid cycle in wheat, suggesting that coordinated regulation of energy metabolism and antioxidant metabolism is essential for stress adaptation [26]. Multi-omics studies in rice, cotton and rapeseed have similarly shown that amino acid metabolism, lipid remodeling, organic acid accumulation and flavonoid metabolism are common mechanisms through which plants manage salt stress [24,27,28]. Previous work has indicated that integrating transcriptomic and metabolomic data can effectively uncover differential regulatory networks between salt-tolerant and salt-sensitive genotypes, identifying key candidate genes and metabolites within pathways including glycerolipid metabolism, phenylalanine metabolism, glycerophospholipid metabolism, amino acid metabolism, glutathione metabolism and ABC transporters [8,29].

Despite considerable progress in wheat salt stress research, most studies have focused on single time points, individual tissues, or isolated omics layers. Systematic comparisons of temporal transcriptional regulation and metabolic reprogramming in roots of wheat genotypes with contrasting salt tolerance during early stress stages remain limited. In particular, the mechanisms by which salt-tolerant cultivars coordinate antioxidant defenses, osmotic adjustment, membrane lipid remodeling, transcription factor regulation, and amino acid metabolism during initial stress to establish robust tolerance have yet to be elucidated. In this study, we compared physiological, transcriptomic, and metabolomic differences between two wheat varieties, LJ114 (salt-tolerant) and CS (salt-sensitive), under salt stress. LJ114 exhibited stronger salt tolerance than CS, as reflected by less severe growth inhibition, stronger induction of antioxidant enzymes, greater accumulation of proline and soluble sugars, and lower MDA content under saline conditions. Multi-omics analysis identified candidate genes and key metabolic pathways related to salt tolerance, involving antioxidant defense, ion transport, energy metabolism, amino acid metabolism, and phenylpropanoid biosynthesis. This study provides novel insights into the molecular mechanisms underlying salt tolerance in wheat and is of great significance for improving salt tolerance in crops.

## 2. Results

### 2.1. Phenotypic and Physiological-Biochemical Responses of LJ114 and CS Roots Under Salt Stress

**Figure 1:**
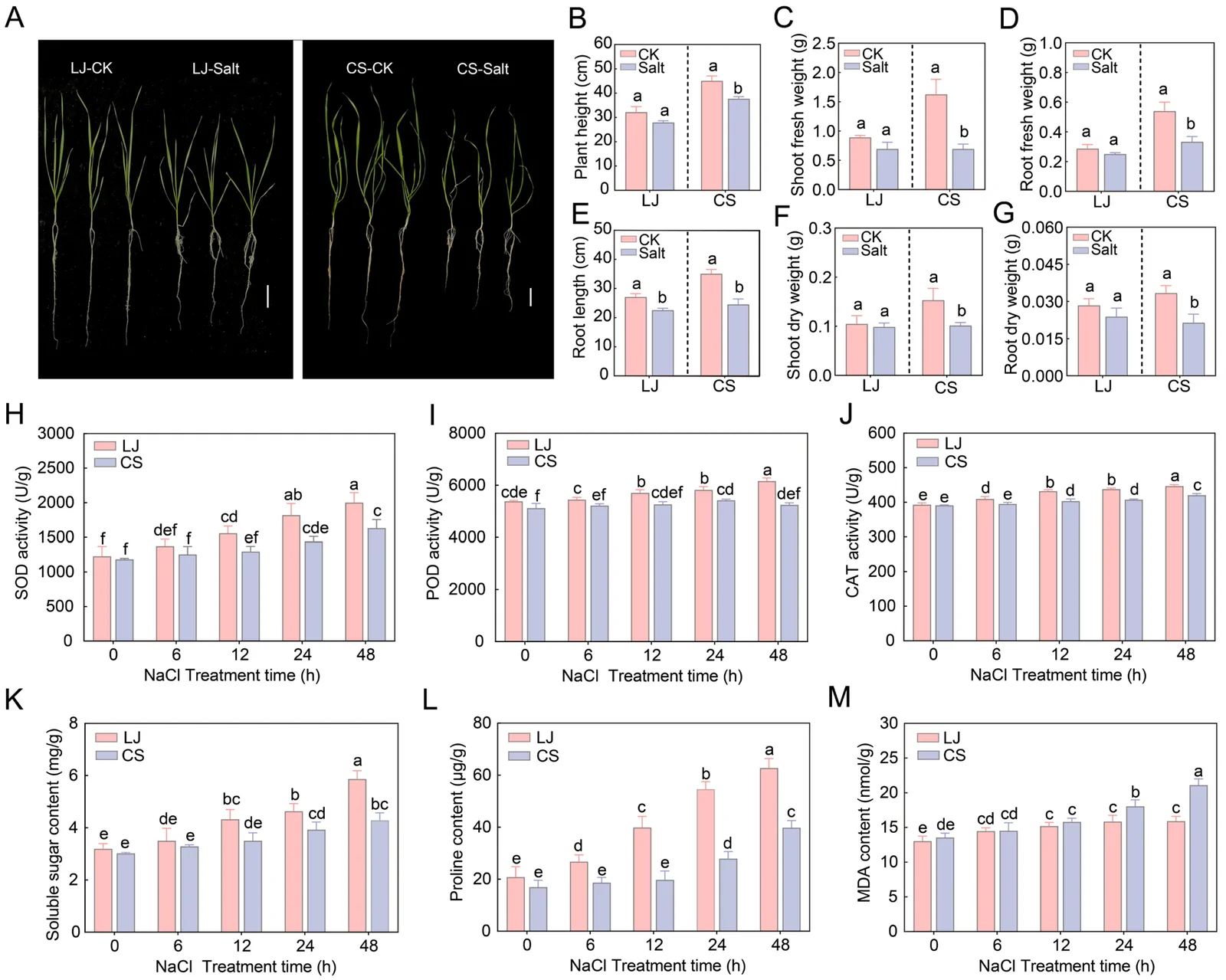
Effects of salt stress on phenotypic, physiological, and biochemical parameters in two wheat cultivars. LJ, Longjian 114; CS, Chinese Spring; CK, control (0 mM NaCl); Salt, 200 mM NaCl. (**A**) Phenotypic changes under control and salt stress. Scale bar, 5 cm. (**B**–**G**) Phenotypic parameters. (**H**–**J**) Activities of superoxide dismutase (SOD), peroxidase (POD), and catalase (CAT). (**K**) Soluble sugar content. (**L**) Proline content. (**M**) Malondialdehyde (MDA) content. Data are mean ± SD of three biological replicates. Different lowercase letters indicate significant differences among treatments, time points, and cultivars (*p* < 0.05).

After 7 d of salt stress, both cultivars showed growth inhibition, but the severity differed markedly. The salt-sensitive CS exhibited severe wilting, with substantially greater leaf chlorosis and curling than LJ114, which maintained a relatively normal growth state (Figure 1A). Relative to controls, CS showed reductions in plant height, root length, shoot fresh weight, shoot dry weight, root fresh weight and root dry weight of 16.36%, 30.04%, 57.38%, 41.60%, 38.27% and 35.86%, respectively; the corresponding decreases in LJ114 were 13.26%, 16.63%, 22.10%, 6.56%, 12.79% and 16.06% (Figure 1B–G, Table S1). Thus, LJ114 showed a smaller reduction range of biomass under salt stress, consistent with stronger salt tolerance.

Antioxidant enzyme activities increased in both cultivars with prolonged stress, and the magnitude of increase was consistently greater in LJ114 than in CS (Table S1). At 12, 24 and 48 h after salt treatment, SOD activity in LJ114 was significantly higher than in CS, increasing by 26.85%, 48.17% and 62.78% relative to 0 h, compared with increases of 12.07%, 28.48% and 37.95% in CS (Figure 1H). POD activity differed significantly between the two cultivars at 6, 12, 24 and 48 h; in LJ114, POD activity increased by 1.36%, 6.20%, 8.18% and 14.50% relative to 0 h, whereas CS showed increases of 1.95%, 2.99%, 5.86% and 2.47% (Figure 1I). For CAT activity, LJ114 increased by 4.20%, 9.25%, 11.46% and 13.71% relative to 0 h, compared with 1.07%, 3.19%, 4.26% and 7.52% in CS (Figure 1J). These results indicate that LJ114 more strongly activates the antioxidant defense system under salt stress.

Salt stress markedly induced accumulation of proline and soluble sugars in both cultivars, with greater accumulation in LJ114. Proline content in LJ114 increased by 28.44%, 91.21%, 162.14% and 201.05% at 6, 12, 24 and 48 h relative to 0 h, compared with increases of 16.37%, 54.86%, 103.72% and 138.29% in CS (Figure 1L). Soluble sugar content in LJ114 rose by 9.62%, 23.14%, 35.36% and 83.63% relative to 0 h, compared with 8.81%, 14.27%, 29.91% and 41.69% in CS (Figure 1K). These data suggest that LJ114 possesses a stronger osmotic adjustment capacity. MDA content increased in both cultivars following salt stress, but the increase was significantly greater in CS than in LJ114 (Figure 1M). The lower MDA accumulation in LJ114 indicates reduced membrane lipid peroxidation and greater cell membrane stability under saline conditions.

### 2.2. Transcriptomic Analysis of the Two Cultivars Under Salt Stress

**Figure 2:**
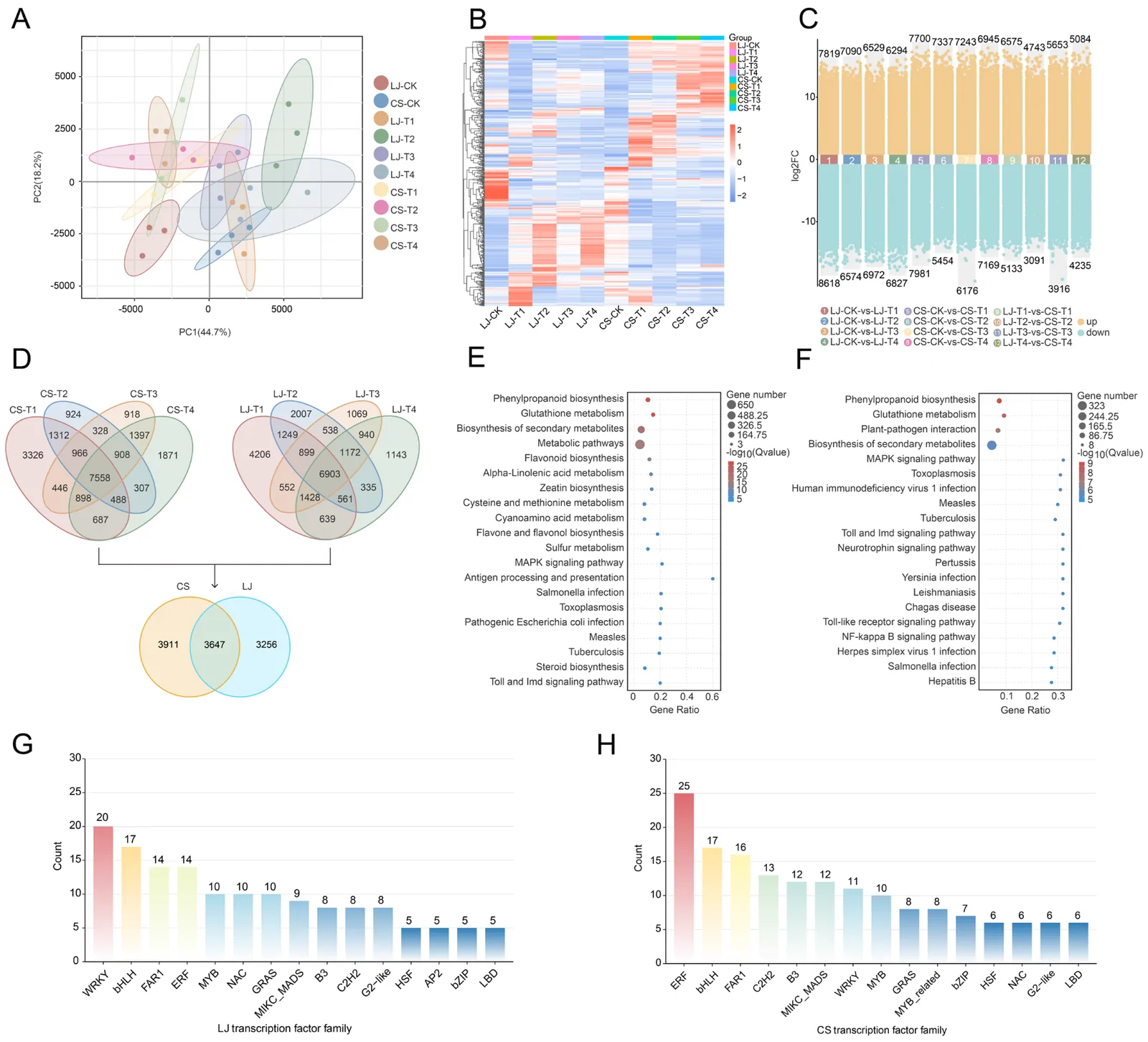
Transcriptome analysis of LJ and CS under salt stress. (**A**) Principal component analysis (PCA) of all gene transcripts. PC1 and PC2, first and second principal components; percentages indicate the proportion of variance explained. (**B**) Heatmap of differentially expressed genes (DEGs). Columns represent treatment groups (LJ, Longjian 114; CS, Chinese Spring; T1-T4, 6, 12, 24, and 48 h of salt treatment). Red, upregulation; blue, downregulation; color intensity reflects magnitude. (**C**) Multi-group differential scatter plot of DEGs. *Y*-axis: log_2_(fold change); points above and below the *x*-axis indicate upregulated and downregulated genes, respectively. (**D**) Venn diagrams showing the numbers of shared and unique DEGs between the two cultivars under salt stress. (**E**,**F**) KEGG enrichment bubble plots for cultivar-specific DEGs in LJ (**E**) and CS (**F**). (**G**,**H**) Transcription factor family distributions among DEGs in LJ and CS.

Transcriptome sequencing of the salt-tolerant LJ114 and the salt-sensitive CS at multiple time points after salt stress yielded 324.29 Gb of clean data (Table S2). Principal component analysis (PCA) of all gene transcripts separated samples by cultivar and treatment time point: PC1 accounted for 44.7% of the total variance, and PC2 accounted for 18.2% (Figure 2A, Table S4). Nine genes were selected to validate their expression levels by qRT-PCR. The results showed that the expression trends were largely consistent with the TPM (Transcripts Per Million) values obtained from transcriptomic analysis, thereby confirming the reliability of the transcriptomic data (Figure S1A–I). Hierarchical clustering confirmed distinct gene expression patterns between the two cultivars across the salt treatment time course (Figure 2B, Table S5).

Differentially expressed genes (DEGs) were identified at 0, 6, 12, and 24 h after salt treatment. In LJ114, the numbers of upregulated DEGs were 7819, 7090, 6529 and 6294 at successive time points, and downregulated DEGs numbered 8618, 6574, 6972 and 6827, respectively. In CS, upregulated DEGs numbered 7700, 7337, 7243 and 6945; downregulated DEGs numbered 7981, 5454, 6176 and 7169, respectively (Figure 2C). Thus, LJ114 showed a higher total number of DEGs than CS during the early stress period (0–6 h), whereas the difference narrowed at 12–24 h, with CS surpassing LJ114 at 24 h. These temporal dynamics reveal distinct patterns of gene regulatory activation between the two cultivars.

Venn diagram analysis showed that 6503 and 7558 DEGs were common across time points in LJ114 and CS, respectively, whereas cultivar-specific DEGs numbered 3256 in LJ114 and 3911 in CS (Figure 2D, Table S6). KEGG enrichment of cultivar-specific DEGs identified common enrichment in phenylpropanoid biosynthesis and glutathione metabolism. LJ114-specific enrichments included flavonoid biosynthesis, alpha-linolenic acid metabolism, and zeatin biosynthesis; CS-specific enrichments involved plant-pathogen interaction and the MAPK signaling pathway (Figure 2E,F). GO enrichment analysis revealed that the differentially expressed genes in the two wheat varieties were mainly enriched in metabolic processes, cellular processes, binding activity, catalytic activity, and cell/membrane-related components (Figure S2A,B). Analysis of transcription factor families revealed that the top five families by DEG count in LJ114 were WRKY, bHLH, FAR1, ERF, and MYB, whereas in CS they were ERF, bHLH, FAR1, C2H2, and B3 (Figure 2G,H; Table S7). Notably, the WRKY family was more abundant among DEGs in LJ114, whereas ERF members were proportionally more abundant in CS.

### 2.3. WGCNA

**Figure 3:**
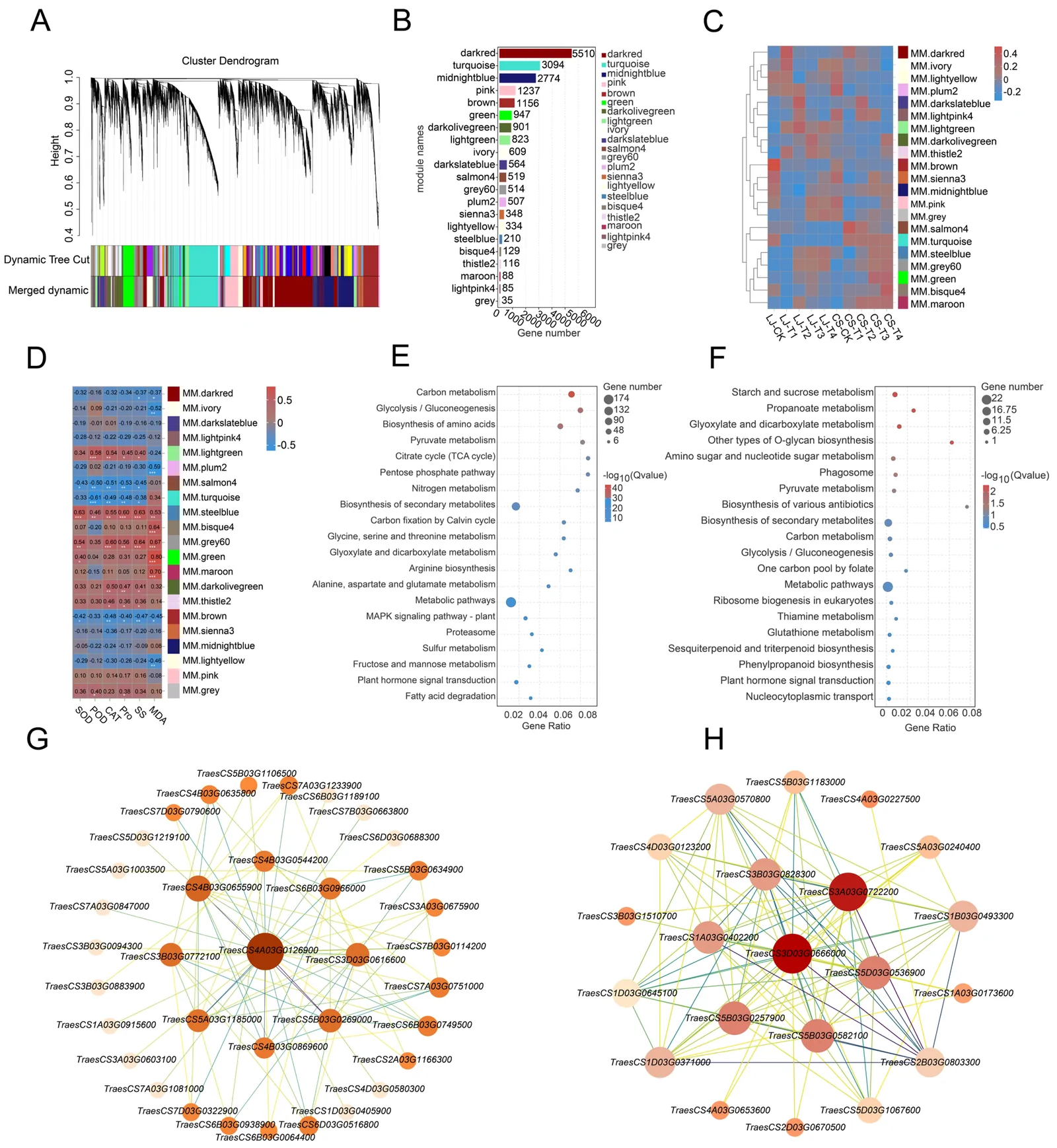
Differential gene co-expression network analysis under salt stress. (**A**) Hierarchical clustering dendrogram of gene co-expression modules. Branches of the same color represent one module. (**B**) Gene counts per module. (**C**) Module-sample expression heatmap. Red, high expression; blue, low expression. (**D**) Trait-module correlation heatmap. Numbers indicate Pearson correlation coefficients. Red, positive correlation; blue, negative correlation. *, *p* < 0.05; **, *p* < 0.01, ***, *p* < 0.001. (**E**,**F**) KEGG enrichment bubble plots for the green module (**E**) and the steel blue module (**F**). (**G**,**H**) Co-expression networks of the top 1% connectivity core genes in the green module (**G**) and the steel blue module (**H**).

Weighted gene co-expression network analysis of transcriptomic data identified 21 co-expression modules after merging similar modules. The dark red module contained the largest number of genes, and the grey module the fewest (Figure 3A,B, Table S8). Module-sample correlation analysis showed that LJ-T1 correlated most strongly with the dark red module, whereas CS-T1 correlated most strongly with the salmon4 module (Figure 3C). Among physiological traits, the green module showed the strongest correlation with MDA content, and the steel blue module showed the strongest correlations with SOD activity and soluble sugar content (Figure 3D).

KEGG enrichment analysis revealed that genes in the green module were primarily enriched in carbon metabolism, glycolysis/gluconeogenesis, biosynthesis of amino acids, pyruvate metabolism, and the MAPK signaling pathway (Figure 3E). Genes in the steel blue module were enriched in starch and sucrose metabolism, propionate metabolism, pyruvate and dicarboxylate metabolism, carbon metabolism, and glutathione metabolism (Figure 3F).

Genes ranked in the top 1% of intra-module connectivity were designated as hub genes, and their co-expression networks were visualized with Cytoscape (Figure 3G,H, Table S9). Hub genes in the green module included *TraesCS4A03G0126900*, *TraesCS4B03G0655900*, *TraesCS1D03G0405900* and *TraesCS3A03G0603100*, with annotated functions in antioxidant defense, carbon and nitrogen metabolism, hormone signal transduction and ion transport. Hub genes in the steel blue module included *TraesCS2B03G0803300*, *TraesCS3A03G0722200* and *TraesCS3B03G1510700*, with annotations implicating roles in cell wall homeostasis and cellulose biosynthesis, secondary metabolism and antioxidant defense, and regulation of energy and carbon metabolism.

### 2.4. Metabolomic Differences Between the Two Cultivars Under Salt Stress

**Figure 4:**
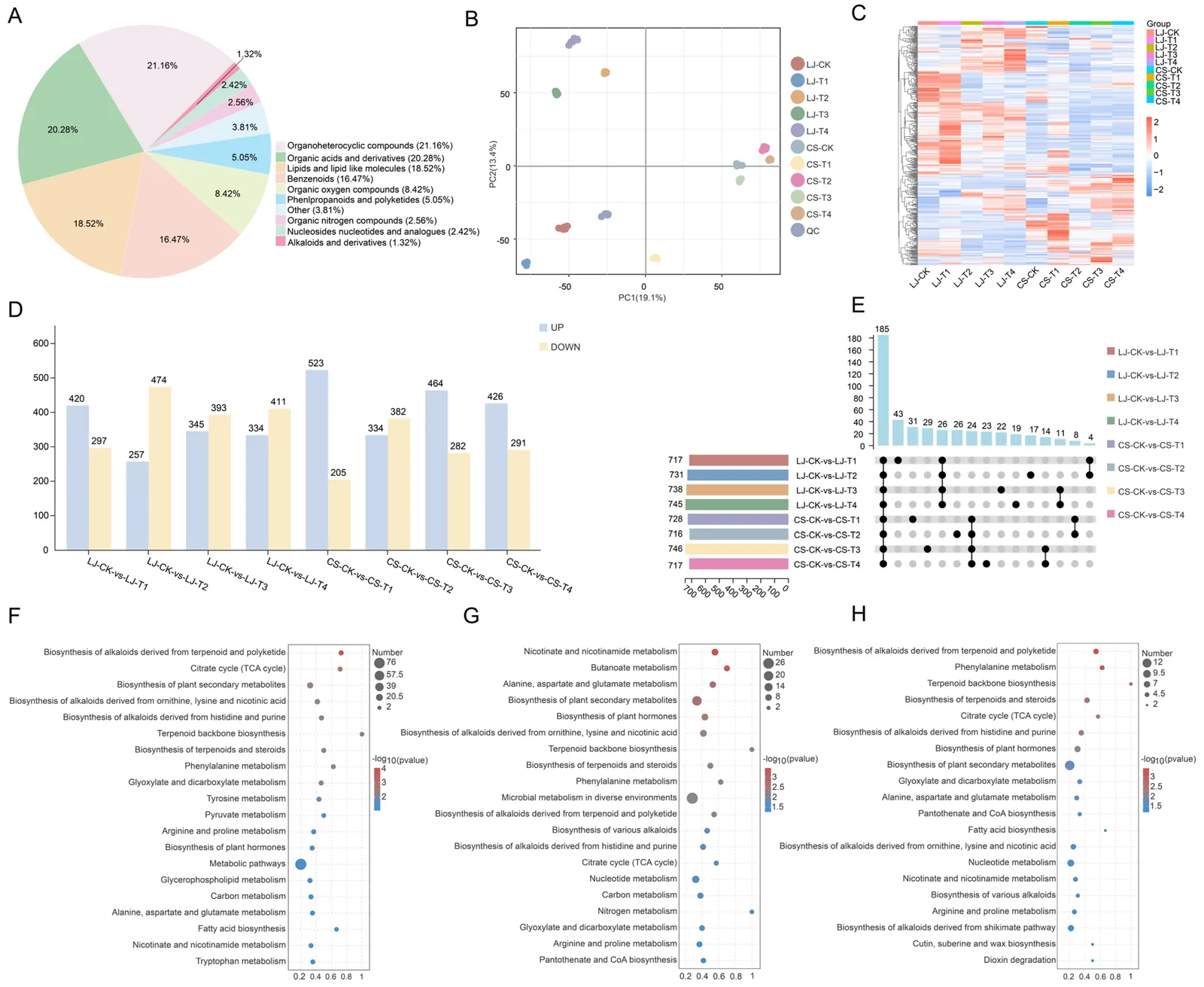
Metabolomic analysis of LJ and CS under salt stress. (**A**) Classification of differentially accumulated metabolites (DAMs). Colors denote metabolite classes. (**B**) PCA of all detected metabolites. PC1 and PC2, first and second principal components. (**C**) Heatmap of DAMs in the two cultivars at different time points. Red, upregulation; blue, downregulation. (**D**) Numbers of DAMs at each time point. Blue, upregulated; yellow, downregulated. (**E**) Upset plot. Solid black dots indicate the comparison groups included in each intersection; bar height indicates the number of metabolites unique to each intersection. (**F**–**H**) KEGG enrichment bubble plots for DAMs common across time points in LJ (**F**), in CS (**G**), and between the two cultivars (**H**). Bubble size represents the number of DAMs per pathway; colors indicate enrichment significance.

Metabolite profiling of roots at 0, 6, 12, 24, and 48 h post-salt treatment revealed 1366 differentially accumulated metabolites between LJ114 and CS. These comprised heterocyclic organic compounds (21.16%), organic acids and derivatives (20.28%), lipids and lipid-like molecules (18.52%), benzenoids (16.47%), organic oxygen compounds (8.42%), and phenylpropanoids and polyketides (5.05%) (Figure 4A; Table S11).

Principal component analysis (PCA) revealed clear separation between the two cultivars along PC1 and PC2, indicating core metabolic differences that were further modulated by treatment duration (Figure 4B, Table S12). Hierarchical clustering confirmed that samples from LJ114 and CS formed distinct clusters with markedly different metabolite expression patterns (Figure 4C, Table S12). The numbers of up- and down-regulated metabolites changed dynamically over time in both cultivars (Figure 4D). During the mid-stage of stress, both cultivars showed a marked increase in down-regulated metabolites. LJ114 reached its peak in total differentially regulated metabolites at the late stage, whereas CS peaked earlier. An UpSet plot showed that 185 differential metabolites were common to both cultivars, with 101 unique to LJ114 and 109 unique to CS across the sampled time points (Figure 4E).

KEGG enrichment analysis of all differential metabolites identified 14 shared pathways between the two cultivars, primarily the tricarboxylic acid (TCA) cycle, carbon metabolism, alanine, aspartate and glutamate metabolism, arginine and proline metabolism, phenylalanine metabolism and biosynthesis of plant secondary metabolites, indicating that salt stress markedly affects energy supply, amino acid metabolism and secondary metabolism in wheat. Differential metabolites unique to LJ114 were specifically enriched in tyrosine metabolism, tryptophan metabolism, glycerophospholipid metabolism, and fatty acid biosynthesis, suggesting more active membrane lipid remodeling and aromatic amino acid regulation under salt stress (Figure 4F). By contrast, CS-specific metabolites were enriched in nucleotide metabolism, nitrogen metabolism, and pantothenate and CoA biosynthesis, reflecting a metabolic response biased toward primary metabolism and coenzyme-related regulation (Figure 4G). Shared differential metabolites between the two cultivars were concentrated in biosynthesis of plant secondary metabolites, phenylalanine metabolism, terpenoid backbone biosynthesis, the TCA cycle, and plant hormone biosynthesis, confirming that reprogramming of energy metabolism, amino acid metabolism, and secondary metabolism is a common feature of the wheat root response to salinity (Figure 4H).

**Figure 5:**
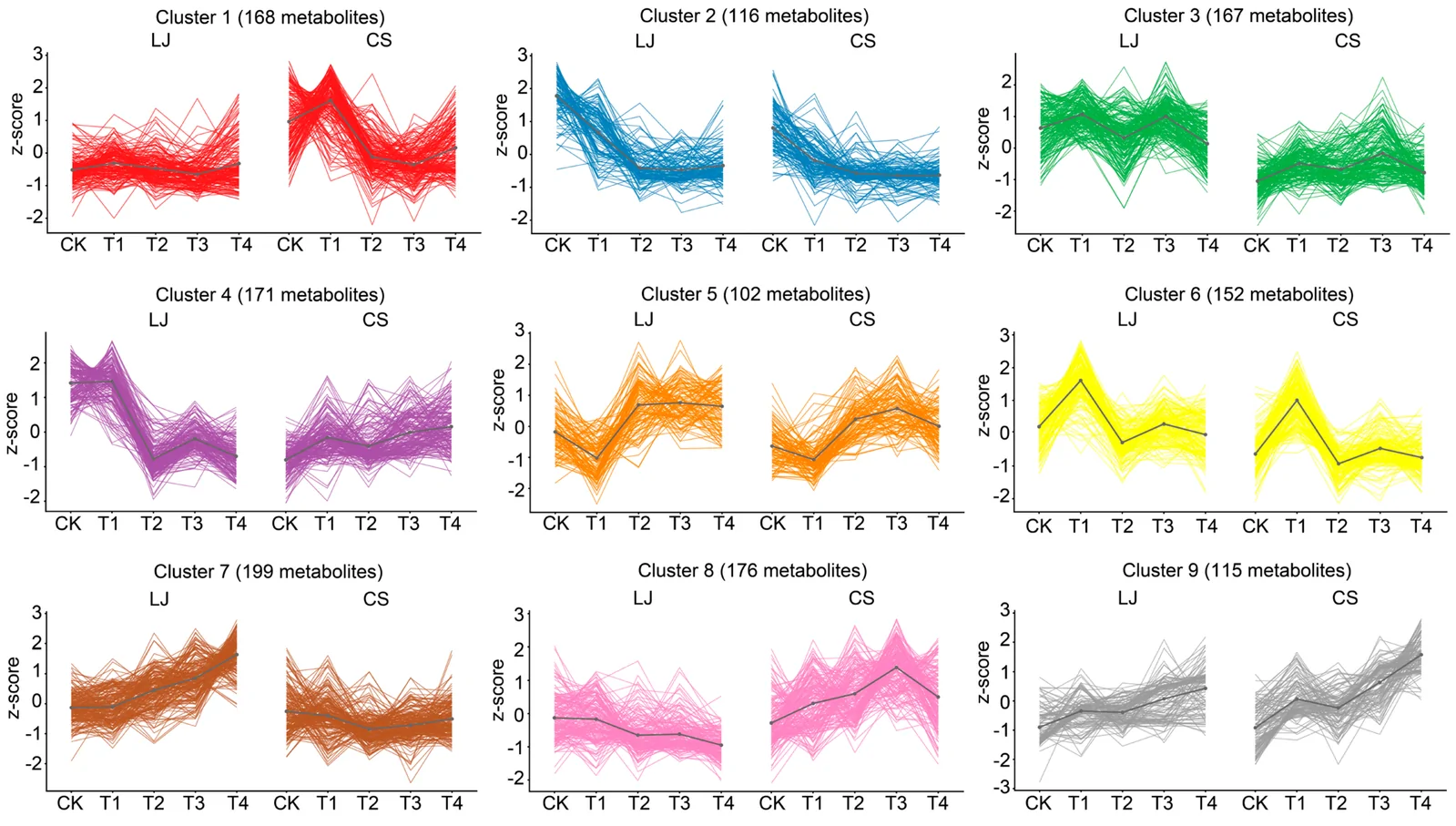
K-means clustering of differential metabolites between LJ and CS. *x*-axis, treatment time points (CK, 0 h; T1-T4, 6, 12, 24 and 48 h of salt treatment); *y*-axis, standardized Z-score of relative metabolite abundance. Black lines represent the temporal expression trend (mean expression pattern) for each cluster.

K-means clustering of the 1366 differential metabolites partitioned them into nine clusters with distinct temporal trends (Figure 5, Table S13). Cluster 9 contained metabolites that were strongly induced by salt stress in both cultivars; these were predominantly phenylpropanoids, heterocyclic organic compounds, and lipids and lipid-like molecules, indicating a conserved set of stress-responsive metabolites. Notably, two LJ114-specific clusters (Clusters 5 and 7) showed substantial metabolite accumulation under salt stress, whereas no obvious changes were observed in CS. Among these, carboxylic acids and their derivatives, organic oxygen compounds, and fatty acyls accumulated markedly with prolonged stress, suggesting that these metabolite classes may contribute to the greater salt tolerance of LJ114.

### 2.5. Integrated Metabolomic and Transcriptomic Analysis Reveals Key Salt-Resistance Metabolic Pathways

**Figure 6:**
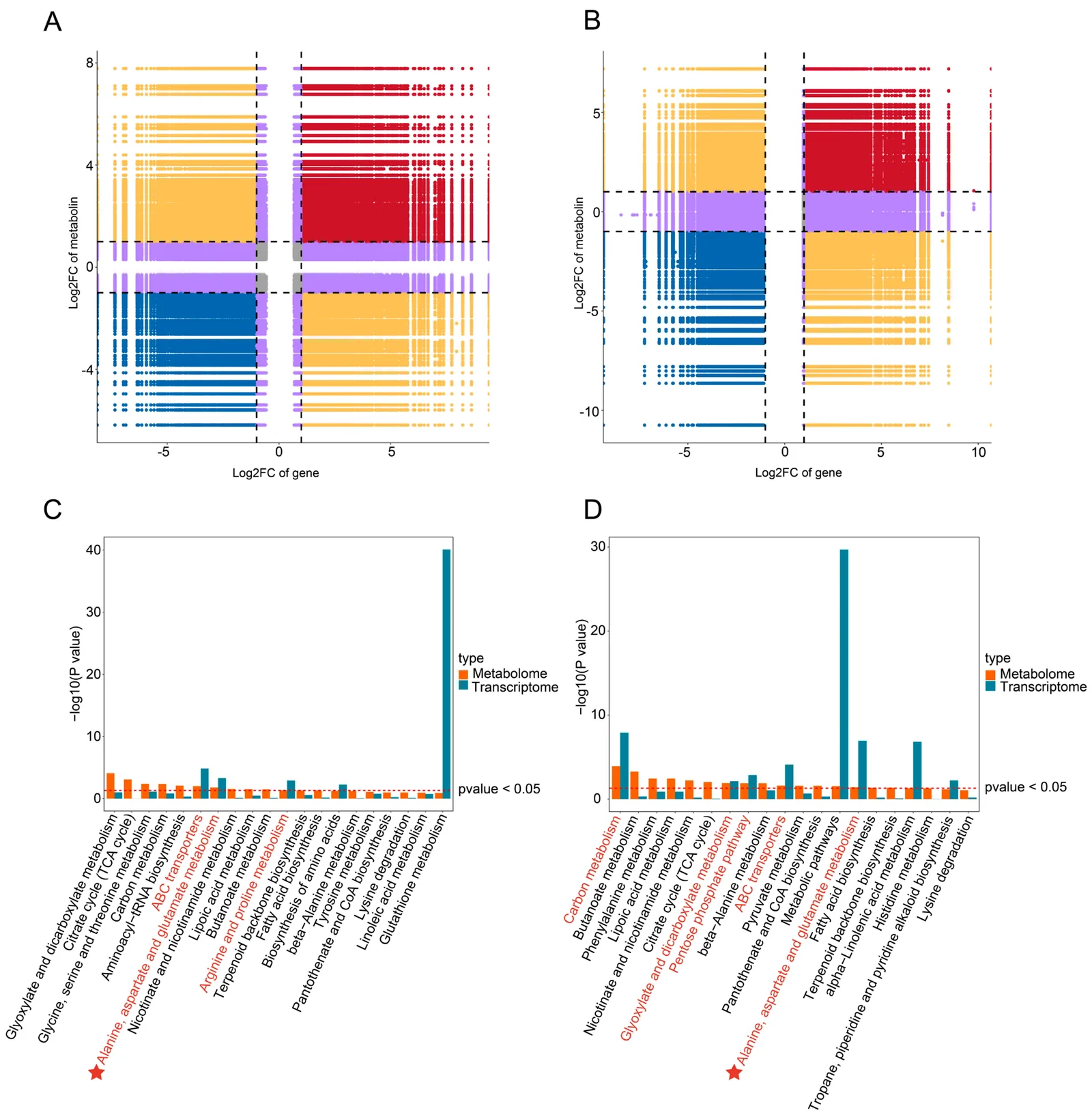
Correlation analysis between transcriptomic and metabolomic data in LJ and CS under salt stress. (**A**,**B**) Nine-quadrant plots of DEGs versus DAMs in LJ (**A**) and CS (**B**). (**C**,**D**) KEGG enrichment bar charts for DEGs (blue) and DAMs (orange) in LJ (**C**) and CS (**D**). Pathways significant in both datasets are highlighted in red, and red stars indicate the pathways that were the main focus of the analysis in this study.

Correlation analysis of differentially expressed genes (DEGs) and differentially accumulated metabolites (DAMs) linked by shared KEGG pathways (Pearson correlation coefficient > 0.8, *p* < 0.05) showed that many gene-metabolite pairs localized to quadrants III and VII of the nine-quadrant plot, indicating coordinated changes in both omics layers (Figure 6A,B). Joint KEGG enrichment of DEGs and DAMs identified the top 20 significantly enriched pathways. In both LJ114 and CS, the ABC transporter pathway and alanine, aspartate and glutamate metabolism were significantly enriched, suggesting that genes and metabolites in these pathways may jointly influence salt tolerance (Figure 6C,D, Table S14). LJ114 showed cultivar-specific enrichment in glutathione metabolism and amino acid biosynthesis, whereas CS showed enrichment in carbon metabolism, pyruvate metabolism, and α-linolenic acid metabolism. These results suggest that the salt-tolerant LJ114 may enhance ROS scavenging and maintain cellular homeostasis through efficient remodeling of antioxidant pathways, whereas CS exhibits broader metabolic reallocation involving carbon metabolism, membrane lipid pathways, and compensatory secondary metabolism.

### 2.6. Analysis of Alanine, Aspartate and Glutamate Metabolic Pathways Under Salt Stress

**Figure 7:**
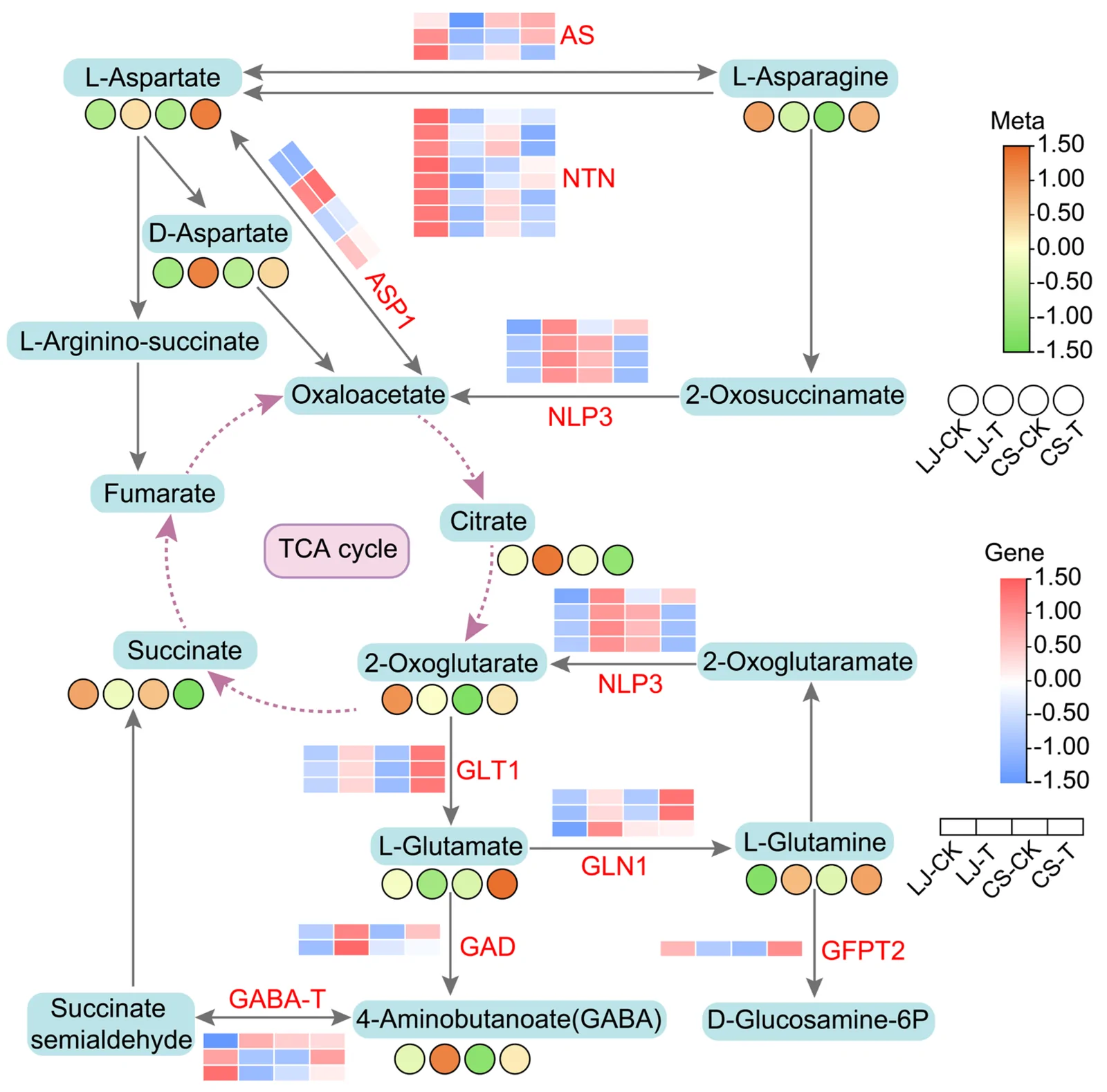
Differentially expressed genes (DEGs) and differentially accumulated metabolites (DAMs) in alanine, aspartate, and glutamate metabolism pathways under salt stress. CK, control; T, 48 h of salt treatment. Orange and green circles represent standardized relative values of DAMs; red and blue cells represent expression levels (TPM) of DEGs. AS, asparagine synthase (glutamine-hydrolysing); NTN, N-terminal nucleophile hydrolase; ASP1, aspartate aminotransferase; NLP3, ω-amidase; GLT1, glutamate synthase; *GAD*, glutamate decarboxylase; GLN1, glutamine synthetase; GFPT2, glutamine-fructose-6-phosphate transaminase; GABA-T, 4-aminobutyrate-pyruvate transaminase.

Integrated transcriptomic and metabolomic analysis revealed cultivar-specific response patterns in the alanine, aspartate and glutamate metabolic pathway. The pathway comprised 33 structural genes with markedly different expression profiles between LJ114 and CS. *ASP1*, *NLP3* and *GAD* showed substantially greater expression changes in LJ114 than in CS. Nine metabolites in the pathway were altered, including L-aspartic acid, L-asparagine, L-glutamic acid, L-glutamine and γ-aminobutyric acid (GABA). Under salt stress, L-aspartic acid, D-aspartic acid, L-glutamine, and GABA were upregulated in both cultivars relative to controls. D-Aspartic acid and GABA were significantly upregulated in LJ114, and the increase in GABA abundance paralleled the expression of its upstream biosynthetic *GAD* genes (*TraesCS4D03G0561600* and *TraesCS3A03G1214200*). Citrate accumulated significantly in LJ114 but decreased in CS. By contrast, L-glutamic acid accumulated significantly in CS with no marked change in LJ114 (Figure 7, Table S15). Together, these results highlight divergent cultivar-specific patterns in both gene expression and metabolite accumulation within this pathway, with LJ114 distinguished by enhanced GABA biosynthesis and citrate accumulation.

## 3. Discussion

This study suggests distinct molecular and metabolic strategies that may be associated with salt tolerance in two wheat cultivars with contrasting sensitivity. By integrating time-series transcriptomics, metabolomics, and physiological measurements in roots of the salt-tolerant Longjian 114 (LJ114) and the salt-sensitive Chinese Spring (CS), we observe that LJ114 exhibits early transcriptional reprogramming, relatively efficient antioxidant defenses and osmotic adjustment, and a tendency for glutamate metabolism to be redirected toward GABA and citrate accumulation. These interconnected features may suggest a proactive stress management strategy, unlike the delayed, compensatory responses in CS.

The physiological superiority of LJ114, reflected in higher SOD, POD, and CAT activities, greater proline and soluble sugar accumulation, and lower MDA levels, is consistent with reports that salt-tolerant maize and wheat varieties mount stronger antioxidant and osmotic responses [30,31,32]. Our temporal transcriptomic data are also in line with the ‘early activation in tolerant, late compensation in sensitive’ model described in other cereal studies [33,34,35]. LJ114 initiates large-scale transcriptional reprogramming within the first 6 h of stress, whereas CS shows a delayed peak at 24 h. This temporal divergence could reflect potential differences in transcriptional and metabolic regulation between the tolerant and sensitive cultivars during the initial response to salt stress.

Both cultivars share enrichment in phenylpropanoid biosynthesis and glutathione metabolism, confirming that cell-wall-associated secondary metabolism and antioxidant systems represent conserved root responses to salinity. However, LJ114 shows preferential enrichment in flavonoid biosynthesis, alpha-linolenic acid metabolism, and zeatin biosynthesis, whereas CS shifts toward plant-pathogen interaction and MAPK signaling, indicating divergent stress signaling strategies. The prominence of WRKY transcription factors in LJ114 and ERF factors in CS further supports this interpretation: WRKY factors have been linked to ROS scavenging and ABA signaling under wheat salt stress [15,36], whereas ERF and MAPK pathway activation in CS may reflect a damage-driven response. Weighted gene co-expression network analysis (WGCNA) identified two functionally distinct modules-a green module correlated with MDA and enriched in carbon metabolism, glycolysis, and MAPK signaling, consistent with an oxidative damage and energy limitation response, and a steel blue module associated with SOD activity and soluble sugar content, enriched in starch/sucrose metabolism and glutathione metabolism, consistent with a protective metabolic module that supports redox and osmotic homeostasis. These network-level distinctions strengthen the biological plausibility of the proposed cultivar-specific strategies.

Metabolomic profiling reveals that wheat salt adaptation comprises both conserved and genotype-specific features. Shared enriched pathways, including the TCA cycle, carbon metabolism, alanine/aspartate/glutamate metabolism, arginine/proline metabolism and phenylalanine metabolism, indicate that energy supply, amino acid turnover and secondary metabolism reprogramming represent common root responses to salinity [8,26]. Genotype-specific enrichment in LJ114 centers on tyrosine metabolism, tryptophan metabolism, glycerophospholipid metabolism and fatty acid biosynthesis, suggesting that this cultivar maintains cellular structural and signaling homeostasis through membrane lipid remodeling and aromatic amino acid metabolism. By contrast, CS shows preferential enrichment in nucleotide metabolism, nitrogen metabolism and pantothenate/coenzyme A biosynthesis, pointing toward basic metabolic compensation and repair. These differences imply that salt tolerance is reflected not only in response magnitude but also in the qualitative allocation of metabolic pathways: LJ114 prioritizes homeostasis maintenance and adaptive remodeling, whereas CS invests in post-stress metabolic remediation.

Integrated analysis identifies ABC transporters and alanine/aspartate/glutamate metabolism as conserved hubs, with LJ114 showing stronger transcriptional responses of ASP1, NLP3, and *GAD* and significant accumulation of GABA and citrate. Citrate accumulation in LJ114 indicates sustained TCA cycle intermediate supply, supporting energy metabolism and carbon skeleton balance under stress [37]. By contrast, L-glutamate accumulation in CS may reflect imbalanced nitrogen metabolism or impaired feedback regulation. Collectively, these findings point to the alanine-aspartate-glutamate pathway as a potential metabolic node linking gene expression reprogramming to salt tolerance. Consistent with reports that GABA enhances antioxidant capacity, alleviates membrane damage and improves growth in wheat [38], LJ114 appears to redirect glutamate metabolism from substrate accumulation toward physiologically functional use, namely GABA synthesis and citrate accumulation-thereby improving carbon-nitrogen metabolic coordination and redox regulation. Overall, salt tolerance may thus involve optimization of metabolic coordination rather than mere changes in metabolite abundance. Nevertheless, several limitations should be acknowledged. Our 200 mM NaCl hydroponic treatment, focusing on early transcriptomic and metabolomic responses, cannot fully reflect long-term adaptations to gradual field salinization, and thus extrapolation to field conditions should be made with caution. Additionally, we did not measure Na^+^ and K^+^ contents in roots and shoots, which prevents us from distinguishing whether the salt tolerance of LJ114 is attributable to ion exclusion or homeostasis. Moreover, candidate genes and metabolites identified through correlative analyses require functional validation via qRT-PCR, transgenic or gene-editing approaches, and exogenous metabolite treatments. Future studies integrating ionomics, proteomics, and functional genomics will be necessary to construct a more comprehensive salt-tolerance regulatory network.

## 4. Conclusions

**Figure 8:**
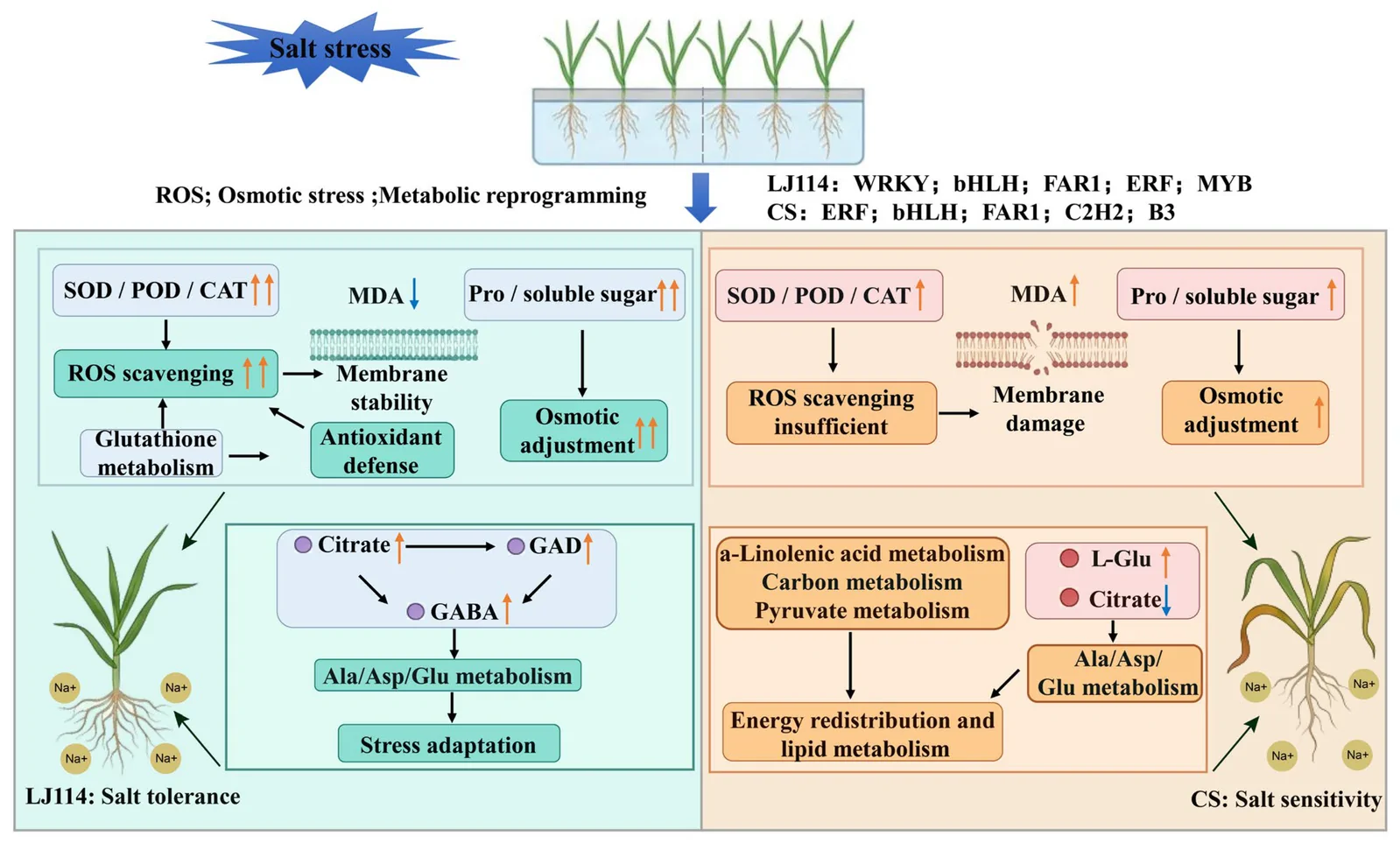
Differences in adaptive mechanisms of wheat in response to salt stress. Orange arrows indicate an increase in accumulation, and blue arrows indicate a decrease in accumulation.

In this study, we compared phenotypic, transcriptomic, and metabolomic differences between wheat accessions LJ114 and CS under salt stress. LJ114 exhibited stronger salt tolerance than CS. Integrated transcriptomic and metabolomic analyses suggested regulation of the alanine, aspartate, and glutamate metabolic pathway as a potential regulatory node linking transcriptional reprogramming to salt-tolerant phenotypes and highlighted specific targets, including *GAD*, GABA, and citrate metabolism, for further functional characterization (Figure 8). Collectively, the presented results provide substantial insight into the molecular underpinnings of wheat responses to salt stress, thereby facilitating the generation of improved salt-tolerant wheat cultivars.

## 5. Materials and Methods

### 5.1. Plant Materials and Growth Conditions

Two wheat varieties were studied: the salt-tolerant Longjian 114 (LJ114) [39] and the salt-sensitive Chinese Spring (CS) [40]. The experiments were conducted at the State Key Laboratory of Aridland Crop Science. Seeds were surface-sterilized in 5% (*v*/*v*) NaClO for 20 min, rinsed five times with sterile water, and germinated in germination boxes lined with moist filter paper at 25 °C for 7 d. Seedlings were transplanted into 24-well hydroponic trays (10 L) containing half-strength Hoagland nutrient solution and grown in a controlled-environment chamber. The environmental conditions of the chamber were a 16 h light/8 h dark photoperiod, day/night temperatures of 25/20 °C, 60% relative humidity, and a light intensity of 400 μmol·m^−2^·s^−1^. The nutrient solution was aerated continuously and refreshed every 3 d.

### 5.2. Sample Collection and Phenotypic and Physiological Measurements

At the three-leaf one-heart stage, seedlings were treated with 200 mM NaCl [41] for 0, 6, 12, 24, and 48 h. The activities of superoxide dismutase (SOD), peroxidase (POD) and catalase (CAT), as well as the contents of malondialdehyde (MDA), proline and soluble sugars in the roots of the two wheat varieties, were measured using commercial kits (Boxbio, Beijing, China) according to the manufacturer’s instructions [42]. Each parameter was assayed in triplicate, and data are presented as mean ± standard deviation. Meanwhile, the plant height, root length, shoot fresh weight, shoot dry weight, root fresh weight, and root dry weight were measured for seedlings treated with 200 mM NaCl for 7 d. Seedlings grown in standard Hoagland nutrient solution served as controls (CK). Three biological replicates were established in each experiment. In addition, the roots of different wheat varieties were collected following treatment with 200 mM NaCl for 0, 6, 12, 24, and 48 h, then frozen in liquid nitrogen and stored at −80 °C for subsequent transcriptomic and metabolomic analyses.

### 5.3. Transcriptome Sequencing and Analysis

Total RNA was extracted from root samples collected at 0, 6, 12, 24, and 48 h after salt stress using the RNAsimple Total RNA Kit (DP419, Tiangen Biotech (Beijing) Co., Ltd., Beijing, China)). RNA concentration, purity, and integrity were assessed using a NanoPhotometer (Implen GmbH, Munich, Germany), a Qubit 2.0 fluorometer (Invitrogen, Carlsbad, CA, USA), and an Agilent 2100 Bioanalyzer (Agilent Technologies, Santa Clara, CA, USA). [43]. Samples with RNA integrity number (RQN) ≥ 5.5 were used for library construction. mRNA was enriched using magnetic beads and fragmented at high temperature, followed by first-strand cDNA synthesis and second-strand synthesis with end-repair and A-tailing. Adapters were ligated, and the products were purified with magnetic beads and amplified by PCR. Libraries that passed quality control were sequenced on an Illumina platform (Illumina, Inc., San Diego, CA, USA).

Raw reads were processed with fastp to remove adapter-containing reads, reads with >10% ambiguous bases, homopolymeric reads, and low-quality reads (Q ≤ 20 in >50% of bases) [44]. Clean read statistics are provided in Table S1. Clean reads were aligned to the wheat reference genome IWGSC RefSeq v2.1 using HISAT2 after removal of residual rRNA sequences with Bowtie2 [45,46]. Specifically, the average total mapping rates were 96.29% for Chinese Spring and 94.90% for LJ114. Transcripts were assembled with StringTie [47], and gene expression levels were quantified with RSEM and normalized as transcripts per million (TPM). Differentially expressed genes (DEGs) were identified using DESeq2 with thresholds of |log_2_ fold change| > 1 and false discovery rate (FDR) < 0.05. Identified DEGs were subjected to KEGG pathway enrichment analysis (www.kegg.jp). Weighted gene co-expression network analysis (WGCNA) was performed to construct co-expression regulatory networks of salt tolerance-related genes, and the resulting networks were visualized with Cytoscape v3.9.1.

### 5.4. qRT-PCR Validation

To validate the transcriptome sequencing results, nine differentially expressed genes were randomly selected for quantitative real-time PCR (qRT-PCR). Primers were designed and synthesized by Sangon Biotech (Shanghai, China), and *TaGAPDH* was used as the reference gene (Table S3). qRT-PCR was performed using the SuperReal PreMix Plus (SYBR Green) kit (Tiangen Biotech (Beijing) Co., Ltd., Beijing, China) according to the manufacturer’s instructions, and relative expression levels were calculated using the 2^−ΔΔCt^ method. Each sample included three biological replicates and three technical replicates.

### 5.5. Metabolomic Analysis

Root samples for metabolomic analysis were collected following the same protocol as for transcriptomic analysis, with six biological replicates. Metabolite separation was performed on an Agilent 1290 Infinity ultra-high-performance liquid chromatography (UHPLC) system equipped with a HILIC column. Samples were injected in randomized order with interspersed quality control (QC) samples to monitor system stability [48]. Mass spectrometric detection was conducted on an AB Triple TOF 6600 mass spectrometer (SCIEX, Framingham, MA, USA) in both positive and negative ionization modes. Raw data were converted using ProteoWizard (version 3.0.22349), and peak detection, alignment, and correction were performed with XCMS. Ion features with >50% missing values or relative standard deviation >50% in QC samples were removed; remaining missing values were imputed using the k-nearest neighbors (KNN) algorithm. Metabolite identification was performed using two complementary approaches: matching against our in-house library of authentic standards and searching public databases (MassBank, METLIN, and MoNA). Both strategies relied on high-accuracy *m*/*z* and MS/MS spectra, with a precursor mass tolerance set at <10 ppm [49]. The identified metabolites were normalized by the sum of peak areas across all samples. Orthogonal partial least squares discriminant analysis (OPLS-DA) was used to distinguish metabolic profiles between groups [50]. Differentially abundant metabolites (DAMs) were selected based on Variable Importance in Projection (VIP) > 1.0, |log_2_(fold change)| ≥ 0.263, and *p* < 0.05 [51]. DAMs were annotated for metabolic pathways using the KEGG database. Pathway enrichment analysis was performed with the hypergeometric test, and FDR correction (Q-value ≤ 0.05) was applied to identify the key metabolic pathways.

### 5.6. Statistical Analysis

All experimental data were analyzed using SPSS 26.0. Two-way analysis of variance (ANOVA) with cultivar and time point as factors was used to assess significant differences, followed by Fisher’s least significant difference (LSD) test for multiple comparisons (*p* < 0.05). Student’s *t*-test was used to compare differences between the two cultivars at the same time point (*p* < 0.05 and *p* < 0.01).

## Data Availability

All relevant data are included within the manuscript and its Supporting Materials. The raw sequencing data have been deposited in the NCBI Sequence Read Archive (SRA) under BioProject accession number PRJNA1520355.

## Supplementary Materials

The following supporting information can be downloaded at https://www.mdpi.com/article/10.3390/plants15182783/s1. Figure S1. RT-qPCR analysis of LJ114 and CS samples. The bar chart shows the qRT-PCR values, and the line chart shows the TPM values for the RNA-seq. (A-I) qRT-PCR values of differential genes. Figure S2. GO enrichment analysis of the two wheat varieties under salt stress. (A) GO enrichment analysis of common DEGs in LJ114 under salt stress. (B) GO enrichment analysis of common DEGs in CS under salt stress. Table S1. The phenotypic, physiological, and biochemical data of two wheat varieties. Table S2. Quality of the sequencing data. Table S3. Sequences of primers used for qRT-PCR. Table S4. Principal component analysis (PCA) data for the RNA-seq dataset. Table S5. Expression data matrix for the clustering heatmap analysis. Table S6. Venn diagram statistics of differentially expressed genes (DEGs). Table S7. Statistical Analysis of Transcription Factors. Table S8. Module clustering data for WGCNA dendrogram. Table S9. List of genes involved in the key module interaction network. Table S10. Statistical Analysis of All Differential Metabolites. Table S11. Principal component analysis (PCA) data for metabolomics. Table S12. Metabolomics data matrix for the clustering heatmap analysis. Table S13. Summary of the K-means clustering results. Table S14. KEGG enrichment data matrix for DEGs and DAMs. Table S15. DEGs and DAMs in the pathways of alanine, aspartate and glutamate metabolism.
