# Supplementary material for: Integrated Multi-Omics Analysis Reveals the Involvement of Alanine, Aspartate and Glutamate Metabolism in Wheat Responses to Salt Stress
Source: Plants (Basel). 2026 Sep 11;15(18):2783. doi: 10.3390/plants15182783 (PMC13610470; doi:10.3390/plants15182783)
Supplement: Supplementary file 1 [file plants-15-02783-s001.zip › Supplementary Figure.pdf]

Supplementary Figures

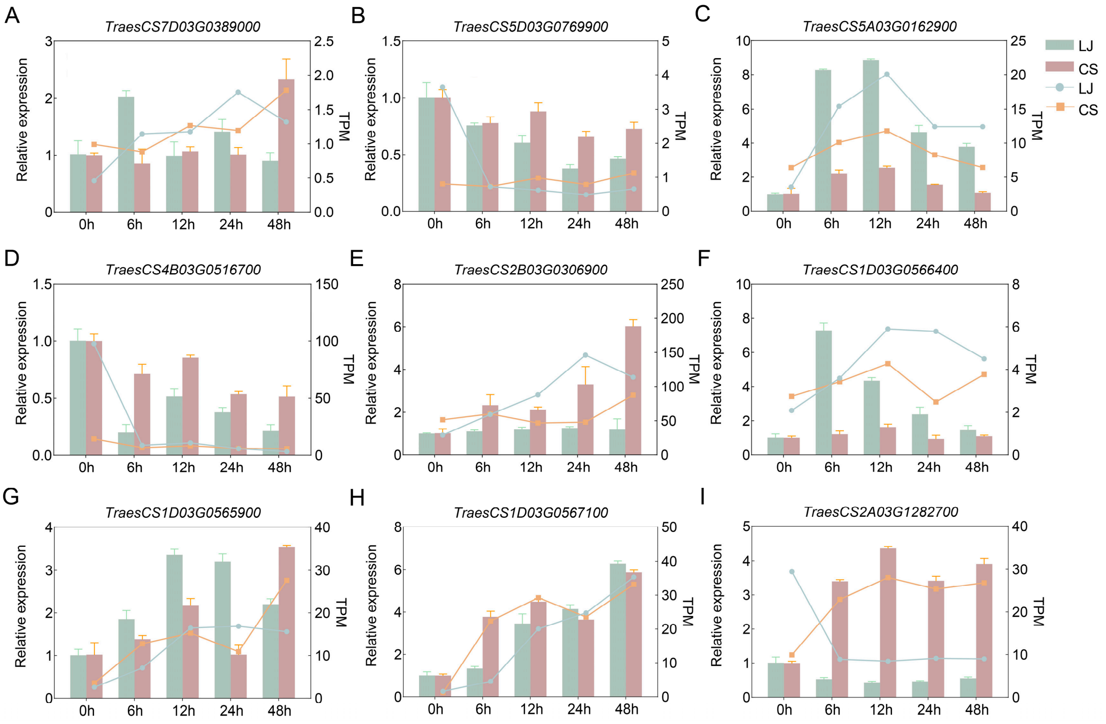

Figure S1. RT-qPCR analysis of LJ114 and CS samples. The bar chart shows the qRT-PCR values, and the line chart shows the TPM values for the RNA-seq. (A-I) qRT-PCR values of differential genes.

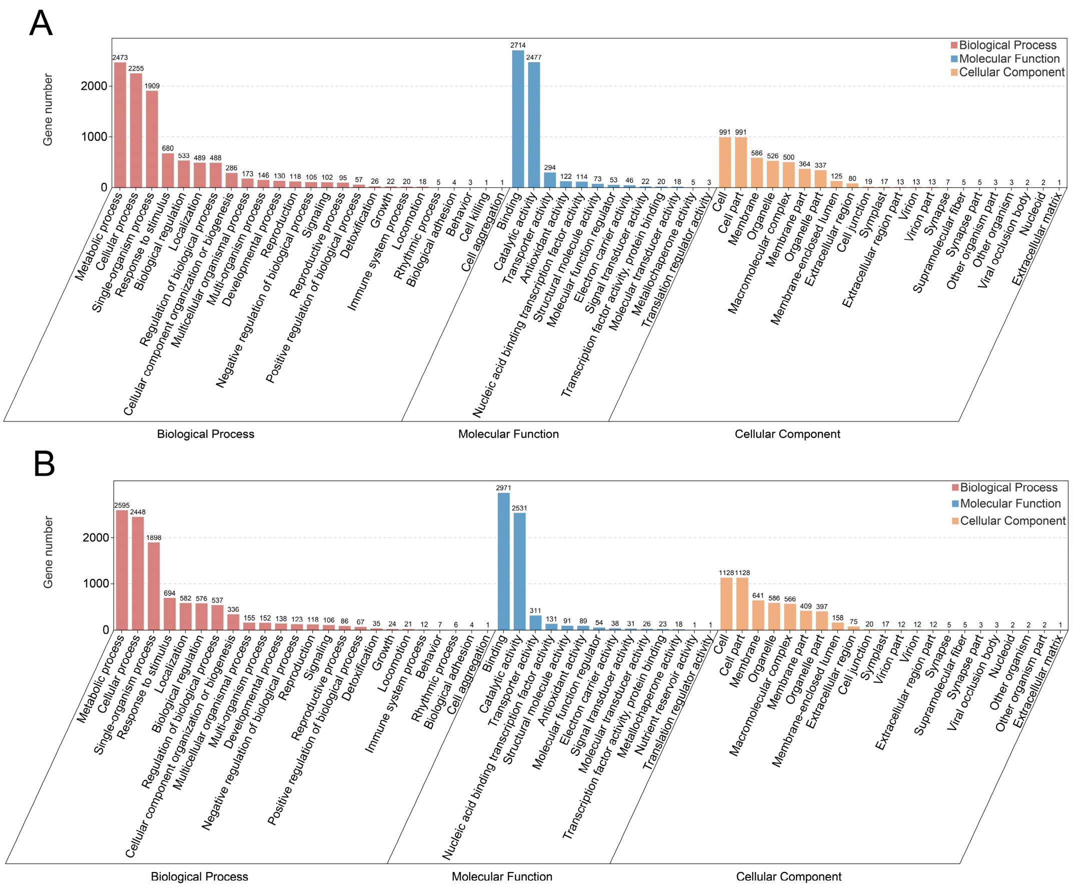

Figure S2. GO enrichment analysis of the two wheat varieties under salt stress. (A) GO enrichment analysis of common DEGs in LJ114 under salt stress. (B) GO enrichment analysis of common DEGs in CS under salt stress.
